# Mitral Valve in Obstructive Hypertrophic Cardiomyopathy: Abnormalities, Management and Controversies

**DOI:** 10.31083/j.rcm2409246

**Published:** 2023-08-30

**Authors:** Zhuheng Wu, Lin Xie, Yajiao Li, Ke Lin, Songbo Zhang, Hong Qian

**Affiliations:** ^1^Department of Cardiovascular Surgery, West China Hospital, Sichuan University, 610041 Chengdu, Sichuan, China; ^2^Department of Cardiology, West China Hospital, Sichuan University, 610041 Chengdu, Sichuan, China; ^3^Department of Surgery, Sichuan Clinical Research Center for Cancer, Sichuan Cancer Hospital & Institute, Sichuan Cancer Center, Affiliated Cancer Hospital of University of Electronic Science and Technology of China, 610041 Chengdu, Sichuan, China

**Keywords:** hypertrophic cardiomyopathy, mitral valve, left ventricular outflow tract obstruction, surgical correction

## Abstract

Obstructive hypertrophic cardiomyopathy (obstructive HCM) is a hereditary disease characterized by septal hypertrophy and dynamic left ventricular outflow tract (LVOT) obstruction. Other than septal hypertrophy, mitral valve abnormalities are also quite common in patients with obstructive HCM, which may contribute to systolic anterior motion (SAM) of the mitral valve and LVOT obstruction. Surgical myectomy is the standard treatment to achieve anatomic correction of obstructive HCM, but controversies remain on whether and how the mitral valve procedures should be performed at the same time. In this review, we first described the mitral valve abnormalities in patients with obstructive HCM and their surgical corrections, we then explained the controversies based on current clinical studies, and we finally made a brief introduction on our surgical strategy and results.

## 1. Introduction

Obstructive hypertrophic cardiomyopathy (obstructive HCM) is a hereditary disease characterized by septal hypertrophy and dynamic left ventricular outflow tract (LVOT) obstruction [1, 2]. Systolic anterior motion (SAM) is the main mechanism for the obstruction of LVOT [2, 3, 4, 5]. Clinical manifestations of obstructive HCM generally include congestive heart failure and arrhythmia [1], and current treatments mainly consist of pharmacotherapy, septal reduction therapy (SRT) and implantation of cardiac defibrillator [6]. Though initial encouraging results have been shown by a newly developed small-molecule drug named Mavacamten, which reversibly inhibits the binding of myosin to actin [7, 8], β-blockers remain the first-line option for pharmacotherapy [6]. Including surgical myectomy and alcohol septal ablation, SRT provides both SAM elimination and symptom improvements, while myectomy is recommended as the standard surgical treatment for obstructive HCM [2].

Mitral valve abnormalities are common in obstructive HCM patients, and it is believed that these abnormalities contribute to SAM and LVOT obstruction in some extent [9, 10, 11, 12, 13], but whether and how mitral valve procedures should be performed during surgical myectomy is controversial [6, 14, 15]. Some HCM centers actively deal with the mitral valve abnormalities [16, 17], while some others hold the opinion that adequate myectomy alone is sufficient to provide satisfactory results [15, 18]. In this review, we first described the mitral valve abnormalities and their surgical corrections in patients with obstructive HCM down to details. Then we summarized current clinical studies to explain the controversies over mitral valve procedures in the surgical correction of obstructive HCM. Finally, we briefly introduced our surgical strategy and results.

## 2. Abnormalities of the Mitral Valve

**Fig. 1.**
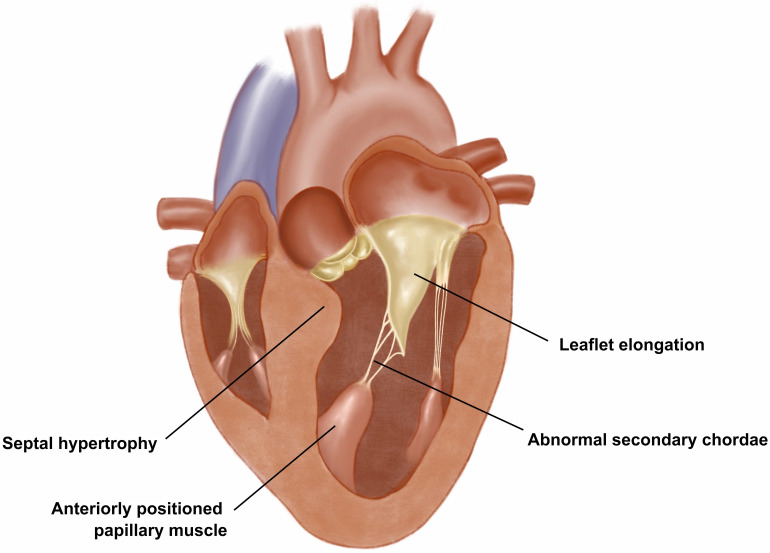
**Abnormalities of the mitral valve in obstructive HCM**. The anterior leaflet of the mitral valve is lax and elongated and is tethered by abnormal secondary chordae. The hypertrophic anterolateral papillary muscle is displaced anteriorly. obstructive HCM, obstructive hypertrophic cardiomyopathy.

In patients with obstructive HCM, the disease process is not confined to cardiac muscle rather many patients also have structural abnormalities of the mitral valve that are unlikely to be acquired or secondary to mechanical factors [9, 10, 11, 12, 13]. According to the abnormal mitral component, abnormalities can be divided as abnormalities of the leaflet, the chordae and the papillary muscle. A single patient may have multiple mitral valve abnormalities, which brings more complexity and uncertainty to the surgical correction [6, 19] (Fig. 1).

Abnormalities of the mitral valve can be evaluated via imaging studies and intraoperative inspection [1]. Preoperative imaging studies include transthoracic echocardiography (TTE) and cardiac magnetic resonance (CMR). TTE is used to assess mitral anatomy, SAM grade and severity of mitral regurgitation, and abnormalities of the leaflet, chords and papillary muscle can be revealed on TTE. CMR can provide comprehensive and precise information on cardiac anatomy and function, mitral abnormalities can be better evaluated by CMR. Intraoperative images are acquired via transesophageal echocardiography (TEE), apart from verifying preoperative results, it also contributes for the evaluation of the surgical outcome. TTE is also performed during follow-up, it monitors the recurrence of SAM and mitral regurgitation.

### 2.1 Abnormalities of the Mitral Leaflet

Mitral leaflet abnormalities mainly include leaflet elongation and increased laxity. Klues *et al*. [9, 10] first conducted an analysis of the leaflet in patients with hypertrophic cardiomyopathy, and they found that increased leaflet length and area are quite common. Subsequent ultrasound and magnetic resonance studies also confirmed the prevalence of mitral valve elongation [20, 21]. Comparing with elongation of the posterior leaflet, elongated anterior leaflet is more common, which averages 31 mm versus 22 mm in controls, and the redundant leaflet tissue beyond the commissure is thought to contribute to SAM [10, 22]. Besides, though rarely seen, elongation of the posterior leaflet is also reported, and the elongated leaflet can lead to SAM and LVOT obstruction [23]. Apart from increased length, increased laxity of the leaflet may also contribute to SAM by its significant deformation during systole [24, 25], and in order to deal with this, some surgeons use a patch to stiffen the leaflet [25, 26, 27].

### 2.2 Abnormalities of the Mitral Chordae

Chordal abnormalities usually present as fibrotic secondary chordae retracting anterior mitral leaflet. In normal individuals, the secondary chordae help to preserve ventricular shape and function during ejection [11, 28]. While in patients with obstructive HCM, secondary chords are often thickened and pull the anterior leaflet towards the septum, thus contributing to SAM [9, 14, 29].

### 2.3 Abnormalities of the Papillary Muscle

Abnormal positioning of the papillary muscle and its insertion directly into the anterior mitral leaflet are the main forms of papillary muscle abnormalities. In patients with obstructive HCM, the anterolateral papillary muscle can be anterior and basilar displaced, thus resulting in the mitral leaflet being more close to the septum [12, 30]. The displacement can be caused not only by the abnormal origin, but also muscular connection between the papillary muscle and the left ventricular free wall [12, 16, 30, 31]. It has been shown in animal modules that anterior displacement of the papillary muscle alone, without septal hypertrophy, can lead to SAM and LVOT gradient [12]. Besides, the anteriorly positioned anterolateral papillary muscle has a higher frequency of bifid malformation [30]. In around 10% of patients with obstructive HCM, direct insertion of the papillary muscle into the anterior leaflet is observed [13, 32, 33]. In this circumstance, other than SAM, the thickened papillary muscle itself can cause LVOT obstruction [32].

## 3. Mechanism of SAM

In patients with obstructive HCM, SAM is the main cause of dynamic LVOT obstruction. In the past, Venturi effect was thought as the dominating mechanism of SAM. It was believed that a pressure differential between the left ventricle (LV) cavity and the LVOT created a suction phenomenon on the mitral leaflet, bringing it toward the septum [34]. However, subsequent studies demonstrated that the velocity of the LVOT was normal at the beginning of SAM, indicating that the Venturi effect was not the initial factor [3, 34]. In fact, the “push” or “drag” effect caused by abnormal blood flow in the LV cavity on the mitral leaflet is the primary effect of SAM. During late diastole and early systole, the hypertrophic septum redirects the flow posteriorly and laterally in the LV cavity, which then strikes the posterior surface of the redundant leaflet, pushing it towards the septum [4, 5]. After mitral-septal contact, the pressure difference itself pushes the obstructing mitral leaflet further into the septum [19]. Although blood flow is the key to SAM, it is undeniable that abnormalities of the mitral valve also contribute to it. So apart from septal myectomy, procedures on the mitral valve may also play an important role in the treatment of obstructive HCM.

## 4. Procedures on the Mitral Leaflet

Procedures focusing on the abnormalities of the mitral leaflet are of great diversity and complexity in the surgical correction of obstructive HCM. Imaging studies before surgery and intraoperative echocardiography are very important for the evaluation and surgical planning of the abnormal leaflet [1, 6]. Redundancy of the leaflet can be corrected through plication and resection, while stiffening can deal with increased laxity. Besides, with the development of interventional techniques, MitraClip has become a potential option for selected patients with unacceptable surgical risk [35, 36].

### 4.1 Leaflet Plication

**Fig. 2.**
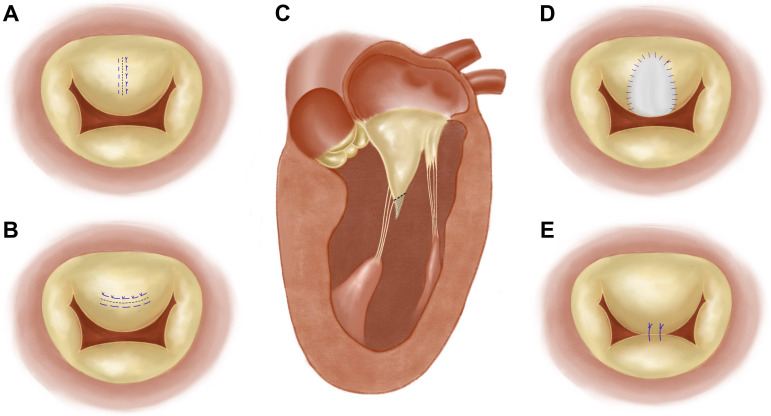
**Procedures on the mitral leaflet**. (A) Vertical plication of the anterior leaflet. (B) Horizontal plication of the anterior leaflet. (C) Partial excision of the anterior leaflet. (D) Extension of the anterior leaflet with a pericardial patch. (E) Edge-to edge repair of the mitral valve.

Plication of the mitral leaflet can be divided into “horizontal plication” and “vertical plication” (Fig. 2A,B). Horizontal plication is plicating the leaflet perpendicular to its long axis, which is a more used technique [16, 17, 37]. After aortotomy and myectomy are performed, 3–5 interrupted horizontal mattress sutures with 5–0 Prolene are placed in the body of the anterior leaflet [16]. The amount of plication is determined by the results of imaging studies and intraoperative inspection, which is usually 2–5 mm [16, 17]. Horizontal plication can effectively decrease the leaflet length, thus preventing the redundant leaflet tissue from being attacked by abnormal blood flow [16, 37]. On the other hand, vertical plication is plicating the leaflet parallel to its long axis, which is usually performed in the A2 section [38]. Different from horizontal plication, this technique focuses on decreasing the leaflet width rather than its length to minish the area exposed to the blood flow [38, 39]. However, this technique may disturb mitral coaptation, causing central mitral regurgitation [19, 40].

### 4.2 Leaflet Excision

Leaflet excision can also decrease leaflet length, it can be performed alone or ancillary to plication [6, 19]. Surgeons should combine the results of imaging studies and intraoperative inspection to decide whether to excise the leaflet and the range of excision. Usually, a segment of A2 is excised for 2–5 mm, and caution must be taken for preventing leaflet flail [19] (Fig. 2C). When obstruction is induced by anterior motion of the posterior leaflet, a part of it should be excised [23]. Normally, the excessive posterior leaflet can be well exposed through aortotomy. If the leaflet is not accessible, a narrow triangular leaflet excision can be performed via a left atrium incision [19].

### 4.3 Leaflet Extension

Other than plication and excision, some surgeons choose to use “leaflet extension” technique, implanting a patch to stiffen the mitral leaflet (Fig. 2D). In this technique, a pericardial patch is harvested after sternotomy and then treated by glutaraldehyde, after which the patch is trimmed to an oval shape approximately 3 cm wide and 2.5 cm long [25]. Then, the anterior mitral leaflet is incised longitudinally from its subaortic hinge point to the rough zone, and the patch is sewn into the leaflet with running Prolene sutures [25]. Though named as “leaflet extension”, this technique mainly widens the leaflet. Possible mechanisms of action of leaflet extension may include: (1) The glutaraldehyde treated patch stiffens the leaflet; (2) Increasing the width of the leaflet erect the relatively lax chordae, both of which make the leaflet less lax and less likely to buckle in the presence of abnormal blood flow [25, 26, 27]. It is worth noting that this technique may further complicate the already complex procedure and elongate the bypass time, thus can bring more potential risk.

### 4.4 Edge-to-Edge Repair

Considering the complexity of the mitral valve anatomy, and for directly limiting the anterior movement of the leaflet, some surgeons combine the transaortic myectomy with Alfieri edge-to-edge repair technique in selected patients with obstructive HCM [41, 42] (Fig. 2E). With the development of interventional techniques, the effectiveness of MitraClip is explored for patients with high surgical risk [35]. The procedure is done under general anesthesia and guidance of transesophageal echocardiography, and only one MitraClip is needed in most cases [36, 43]. Current experience demonstrates that, after MitraClip implantation, patients should have significantly decreased LVOT gradient, reduced mitral regurgitation, SAM elimination and improved New York Heart Association (NYHA) classification [36, 43, 44, 45]. However, most of the evidence in this area is based on case reports or case series, the safety and effectiveness of MitraClip in patients with obstructive HCM need to be further verified.

## 5. Procedures on the Mitral Chordae

**Fig. 3.**
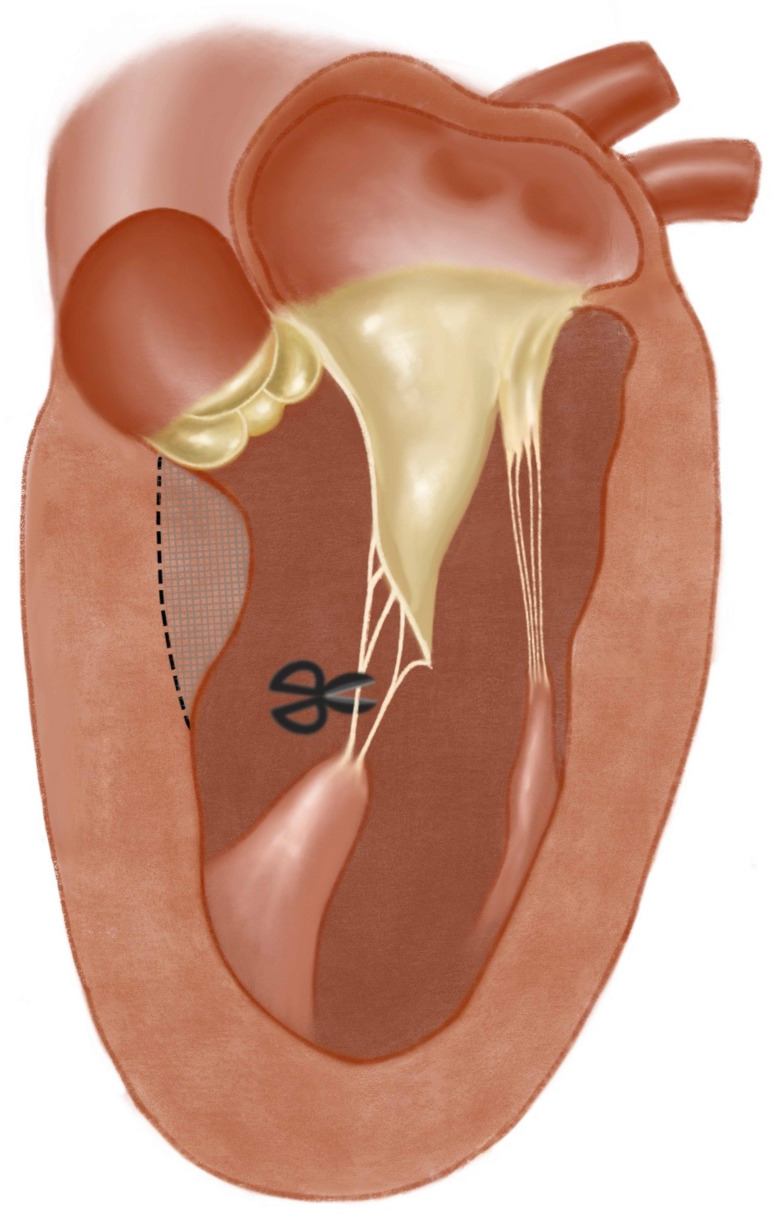
**Resection of the secondary chordae**. The anterior leaflet is tethered by the abnormal secondary chordae, resection of the chordae is performed at the same time of myectomy.

In patients with obstructive HCM, the mitral leaflet can be retracted by the abnormally fibrotic secondary chordae, and chordal resection can let the tethered leaflet fall posteriorly and away from the septum [6, 46] (Fig. 3). Echocardiography and magnetic resonance can identify the abnormal chordae before surgery, and further verification should be made during surgery [1]. Chordal resection can be performed via aortotomy. After myectomy, forceps are sued to push the anterior leaflet towards the left atrium to identify the retracted chordae, then the leaflet end and the papillary muscle end of the chordae are cut to resect it [14]. If thickened fibrotic tissue presents on the chordal attachment site of the leaflet, it should be carefully resected at the same time [14].

## 6. Procedures on the Papillary Muscle

Papillary muscle release and resection are two major forms of papillary muscle procedures in patients with obstructive HCM. Besides, if bifid and hypermobile papillary muscle is present, some surgeons may use pledgeted sutures to perform papillary muscle reorientation [47, 48].

### 6.1 Papillary Muscle Release

**Fig. 4.**
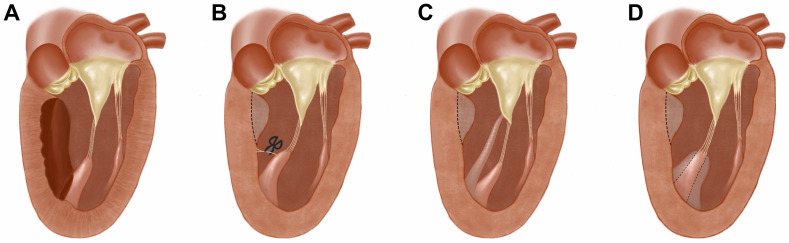
**Procedures on the papillary muscle**. (A) Extending the myectomy beyond the origin of the anterolateral papillary muscle to achieve its release. (B) Resection of the muscular connections between the papillary muscle and the LV wall. (C) Partial resection of the papillary muscle which inserts directly into the mitral leaflet. (D) Longitudinal resection of the papillary muscle (or papillary muscle thinning) to eliminate the obstruction caused by its hypertrophy.

Papillary muscle release can be achieved via extended myectomy, during which the incision is extended laterally and beyond the origin of the anterolateral papillary muscle, making it fall posteriorly [32, 49] (Fig. 4A). For patients whose papillary muscle originated normally, muscular connections between the papillary muscle and the left ventricular free wall may play an important role in the anteriorly positioned leaflet, thus resection of these abnormal connections is reasonable [16, 32, 37] (Fig. 4B). Current evidence also indicates that after papillary muscle release and secondary chordae resection, the mitral and aortic annulus may go back to a more parallel relationship [50, 51].

### 6.2 Partial Resection of the Papillary Muscle

In some situations, in order to release the leaflet, partial resection of the papillary muscle should be performed when it inserts directly into the midportion of the anterior leaflet [32] (Fig. 4C). Another technique of partial resection is called papillary muscle thinning, during which the hypertrophic papillary muscle is resected longitudinally to eliminate its direct obstruction [19, 33] (Fig. 4D).

## 7. Current Controversies

Since the proposition of myectomy and its evolvement, it has become the key to surgical correction of obstructive HCM [52, 53]. However, as studies on mitral abnormalities continue to emerge, whether to perform mitral valve procedures at the same time of myectomy has become quite controversial [6, 14, 15, 54].

In order to deal with septal hypertrophy and mitral abnormalities simultaneously, some surgeons brought up “resect-plicate-release” or “RPR” procedure [40]. In this procedure, a combination of extended septal myectomy, anterior leaflet plication together with papillary muscle release and secondary chordal resection is used to treat all possible mechanisms of dynamic LVOT obstruction [40]. The RPR procedure is logically reasonable, it aims to achieve anatomic correction, and current evidence also shows good results [16, 17, 19, 49]. However, it should be noted that, comparing with extended myectomy alone, the PRP procedure not only lengthens the bypass time, but also increases surgical complexity [55]. Besides, the effectiveness of the mitral valve procedures (such as mitral valve plication) has not been verified [15].

Leading by Mayo Clinic, some centers insist that extended myectomy is enough for the surgical correction of obstructive HCM, and mitral valve procedures should only be performed when intrinsic mitral valve disease is present [15, 18]. A retrospective study from Mayo Clinic which includes 2107 obstructive HCM surgeries indicates that: when there is no intrinsic mitral valve disease, 96.1% of surgical corrections can be achieved via extended myectomy alone and with promising outcomes [15]. It is believed that extended septal myectomy is sufficient to eliminate SAM and LVOT obstruction, and mitral valve procedures only provide a buffer against failure that may occur because of imprecision in depth and extent of myectomy [15, 19]. It must be noted that though Mayo Clinic stated that “we proceeded with extended septal myectomy alone” [15], previous study explaining their surgical techniques mentioned that “Trabeculations and abnormal chordae are excised” during myectomy [56]. So, the definition of “mitral valve surgery” in their study may be closer to “mitral leaflet surgery”. On the other hand, another research from Mayo Clinic reveals that increased anterior mitral leaflet length is not associated with higher LVOT gradient [57]. Comparing with the length less than 30 mm, patients with anterior leaflets longer than 30 mm do not have higher LVOT gradients (49 mmHg vs 50.5 mmHg, *p* = 0.76), and anterior leaflet length also has nothing to do with the postoperative LVOT gradient relief [57]. These results hint that the anterior leaflet plication may not be as effective as we previously thought.

In recent years, another subgroup of patients with obstructive HCM is getting more attention. These patients only have mild septal hypertrophy (<18 mm), but their SAM and LVOT obstruction are quite significant [19]. Due to their relatively thin septum, extended septal myectomy alone may not guarantee a good result, but can lead to a catastrophic septal defect [58]. So in the past, mitral valve replacement is the major solution for these patients [58]. A recent study from Ferrazzi *et al*. [14] shows that a shallow septal myectomy combined with secondary chordal resection come up with good results. It reveals that comparing with myectomy alone, additional secondary chordal resection results in a lower postoperative LVOT gradient and a more significant NYHA classification improvement [14], and these results are then proved by further investigations [59, 60]. Ram *et al*. [50] chose extended septal myectomy combined with secondary chordal resection in nonselective patients with obstructive HCM, and their results indicated that patients who received additional chordal resection had a greater LVOT gradient relief. In all, considering mitral leaflet procedures are complex and time-consuming, extended septal myectomy together with secondary chordal resection may be a better surgical strategy for patients with obstructive HCM.

## 8. Our Choice

We agree that extended septal myectomy is critical in the surgical correction of obstructive HCM, and we also insist that making the mitral leaflet away from the abnormal flow field in the LVOT is also of great importance in SAM elimination. For the consideration that mitral leaflet procedures represented by leaflet plication may bring complexity and uncertainty to the surgical treatment, we choose a combination of extended septal myectomy and secondary chordal resection as the standard surgical treatment for patients with obstructive HCM (Fig. 3). We believe that as a less complex and less time-consuming procedure, secondary chordal resection can release the mitral leaflet and make it fall more posteriorly, thus away from the abnormal flow field in the LVOT.

We retrospectively collected the data of patients with obstructive HCM who underwent septal myectomy with secondary chordal resection at our center, and all patients were operated by a single surgeon. Echocardiographic data presented here were from transthoracic echocardiography performed preoperatively and postoperatively before discharge, and patients’ NYHA classification were acquired preoperatively and 3 months after surgery. Age is presented as mean, and other data are presented as mean ± standard deviation or proportions. Since 2014 to 2020, a total 73 consecutive patients with obstructive HCM received extended septal myectomy with secondary chordal resection in our center. Their mean age was 47.3 years and 54.8% of them were male. Their mean preoperative septal thickness was 21.44 ± 7.1 mm, mean LVOT gradient was 72.26 ± 29.39 mmHg, 79.2% of them had a moderate or severe mitral regurgitation, and 72.6% (52/73) of them had a NYHA classification III and IV. Postoperative mean septal thickness decreased to 13.69 ± 2.95 mm, mean LVOT gradient decreased to 13.32 ± 9.24 mmHg, only 3 patients had a moderate or severe mitral regurgitation (4.1%), and no patient had postoperative SAM. Only 1 patient had postoperative NYHA classification III (1.3%). One patient developed a moderate-to-severe mitral regurgitation due to posterior leaflet prolapse 3 months after surgery.

## 9. Conclusions

In patients with obstructive HCM, other than septal hypertrophy, mitral abnormalities are also quite common and contribute to SAM and LVOT obstruction. In the surgical correction of obstructive HCM, apart from septal myectomy, mitral valve procedures are also been used, but controversies remain on whether and how the mitral valve procedures should be performed. Plenty of surgical techniques can be used to deal with the mitral abnormities, which brings more complexity and uncertainty to the surgical treatment at the same time. Some centers believe that extended myectomy alone can provide good results, besides, they also have doubts on the necessity and effectiveness of the mitral valve procedures, and the latest results from Mayo Clinic also indicate that leaflet plication may not be as effective as we previously thought. However, some centers choose to actively operate on the abnormal mitral valves, and they hold the opinion that correcting the anatomic abnormalities can further ensure SAM elimination and relief of the LVOT obstruction. In recent years, septal myectomy with secondary chordal resection is thought as a potentially better option. Comparing with other mitral procedures, secondary chordal resection is less complicated and less time-consuming. We use this strategy as our first line surgical treatment at our center and it comes up with good results, but we still think that further investigations are needed to verify the safety and effectiveness of this strategy. Finally, we recommend that patients with obstructive HCM should receive surgical treatment in experienced centers, and surgical strategy should be personalized.
